# A 0.5 MP, 3D-Stacked, Voltage-Domain Global Shutter Image Sensor with NIR QE Enhancement, Event Detection Modes, and 90 dB Dynamic Range

**DOI:** 10.3390/s23239448

**Published:** 2023-11-27

**Authors:** Adi Xhakoni

**Affiliations:** ams OSRAM, Borsbeeksebrug 36, 2600 Antwerp, Belgium; adi.xhakoni@ams-osram.com

**Keywords:** image sensors, NIR, global shutter, voltage domain, HDR, AR/VR, eye tracking, ADC, low power

## Abstract

We introduce a compact voltage-domain global shutter CMOS image sensor for a wide range of applications including consumer, IoT, and industrial applications. With 0.5 MP and 2.79 µm pixels packed in a die size of only 2.3 mm × 2.8 mm, the sensor achieves more than 92% quantum efficiency (QE) in the visible wavelength and more than 36% at near-infrared (940 nm) all while drawing a mere 20 mW at a 10-bit, 30-frame-per-second operational mode. In this article, we focus on the architecture of the sensor and the design challenges encountered to fit all the necessary circuitry in such a limited footprint. Moreover, we detail the new solutions we have developed to meet the demanding specifications of low-power operation and high dynamic range (HDR).

## 1. Introduction

**Figure 1:**
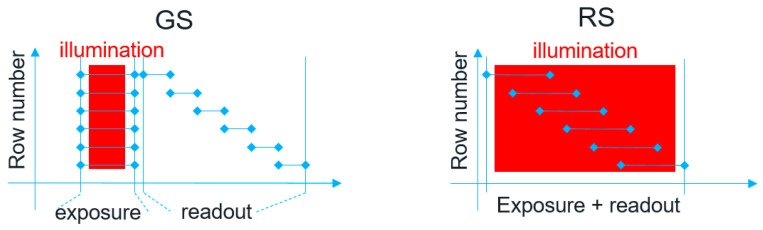
Global shutter sensor (**left**) exposing all pixels at the same time vs. rolling shutter sensor (**right**) exposing rows of pixels sequentially.

Many fields, including machine vision, robotics, consumer, etc., require global shutter image sensors due to the need for image artifact reduction and their capability to synchronize with a pulsed illuminator. The latter is very important for power consumption since in global shutter sensors, all pixels are exposed at the same time, as shown in Figure 1 [1]. In contrast, rolling shutter pixels have rows exposing sequentially, therefore the illuminator needs to stay active until all the rows finish exposing, using unnecessary power, and potentially causing overheating of the system. Some works have proposed using illuminators with addressable light patterns, which follow the row exposure of rolling shutter sensors [2], partially improving the system’s power consumption. However, that comes at the expense of increased complexity and cost of the illuminator and image artifacts caused by fast-moving objects in the scene or camera movement. It is therefore crucial for sensors combined with a pulse illuminator to employ global shutter technology to keep the power consumption and cost of the system under control. Among global shutter technologies, voltage [3] and charge domains [4] are the most used ones. For low power consumption, voltage domain has the edge due to its possibility to be combined with backside illumination [5] and near-infrared (NIR) quantum efficiency enhancement light-scattering structures [6,7,8] while maintaining good parasitic light sensitivity. High QE is crucial for the illuminator to have low power consumption.

Focusing on the target application of the reported design being AR/VR glasses, other key requirements are low system power (to reduce the weight of the battery and/or increase the charging intervals) and miniaturized silicon size (to fit within the thin frame of the glasses) [9]. Furthermore, HDR operation is especially required for world-facing cameras. To summarize, the ideal image sensor for AR/VR consumer applications is a global shutter with low system and sensor power consumption and a very small size.

To our knowledge, the combination of the above-mentioned specs has not been previously reported in a single sensor.

To satisfy those requirements, we designed a 3D-stacked, 0.5 MP image sensor with 2.79 µm voltage-domain GS pixels with a packaged sensor footprint of only 2.3 mm × 2.8 mm. On top of the above features, we added smart event detection modes and single-shot HDR operation.

More details on this sensor are available in the following sections: Section 2 describes the sensor architecture, focusing on the logic layer; Section 3 introduces the high dynamic range technique, and Section 4 talks about the smart event detection mode. Finally, the summary and conclusions are available in Section 5.

This paper is an invited journal extension of our conference paper [6].

## 2. Sensor Architecture

**Figure 2:**
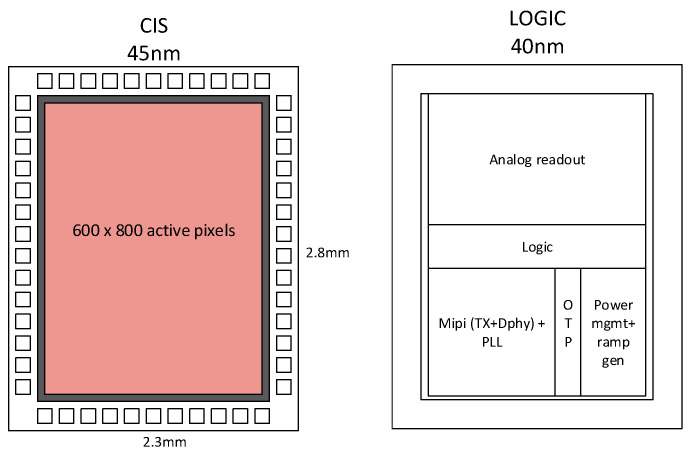
CIS silicon (**left**) stacked on top of logic silicon (**right**) via hybrid bonding interconnects.

To meet the challenging requirements of footprint and power for consumer applications, we designed an image sensor with the architecture depicted in Figure 2. Wafer-to-wafer 3D stacking with high-density hybrid bonding (HB) allows the placement of the backside-illuminated (BSI) pixel array on top of the logic die, which acts like a readout and drives circuitry for the pixel array. The pixel array is connected to the logic die via HB at the column and row level. No per-pixel HB interconnect is necessary since the pixel uses only the top silicon layer. The pixel array uses 45 nm CMOS optimized for optical performance and employs NMOS-only transistors. The logic die is a standard 40 nm CMOS process optimized for low power.

**Figure 3:**
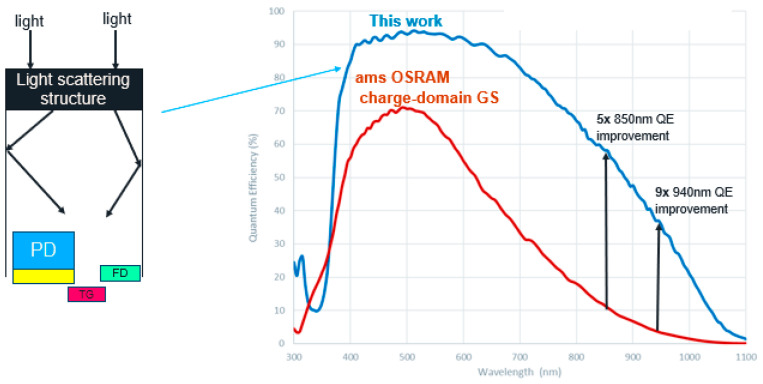
The added light-scattering structure increases QE in both visible and NIR wavelengths.

To increase the QE, we used a special scattering layer on top of the pixel to extend the photon path within the pixel without increasing the EPI thickness (left drawing of Figure 3). Combined with BSI technology, QE levels of more than 90% are achieved in the visible range and >36% in the 940 nm range. A comparison of the QE achieved in this work and that of a charge-domain GS sensor from ams OSRAM is depicted in Figure 3. QE improves by almost an order of magnitude at 940nm (right drawing of Figure 3). The light-scattering structure is quite effective when combined with a voltage-domain global shutter pixel since it increases the QE without impacting the parasitic light sensitivity (PLS) [7,10].

### 2.1. Footprint Reduction

We decided to keep the ADC pitch the same as the pixel pitch to avoid potential column FPN issues. This posed a challenge in the row driver design, as we needed to keep the sensor footprint within specifications. However, by improving the row driver architecture and using seven metal layers made possible by the stacked technology, we were able to achieve an exceptionally narrow row driver width of only 40 µm. The thick last metal in particular made an impressive difference in power distribution across the narrow row driver area. Due to that, we were able to reduce the width of the row driver by more than 10 times compared to our previous sensors using traditional frontside-illuminated (FSI) technology [3,11,12,13]. Additionally, we split the row driver into two parts (left and right of the array) to further reduce its width and to have a more symmetrical placement of the readout blocks.

These improvements in architecture, layout, and technology node scaling allowed us to create a sensor footprint that is mostly dominated by the pixel array rather than readout silicon. This is a significant improvement over previous designs, where the readout silicon could occupy a significant portion of the sensor area [3]. This is even more critical when the sensor resolution decreases since less room is available for the readout circuits, while standard interfaces (e.g., MIPI CSI2) are still expected by the customers/applications.

**Figure 4:**
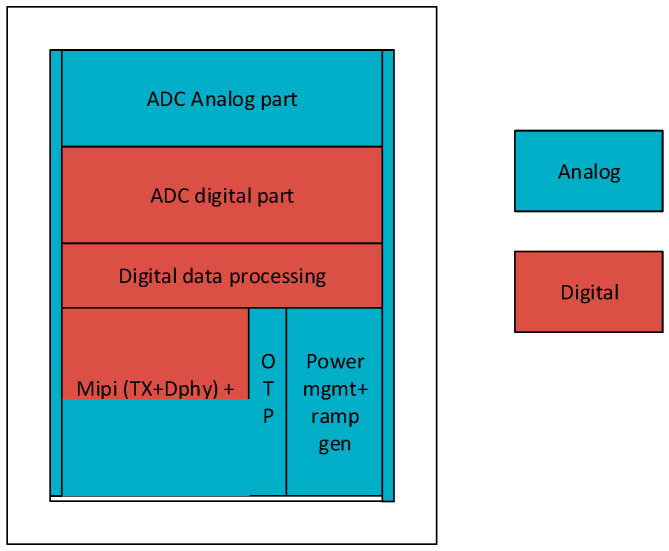
After natural shrinkage of digital blocks from technology node scaling, special layout techniques were used to shrink the row drivers and analog parts of ADC.

Figure 4 depicts the area usage of the logic blocks. Since the digital cells make use of the scaled technology node for power and area reduction, the pressure was on the analog blocks to keep up.

**Figure 5:**
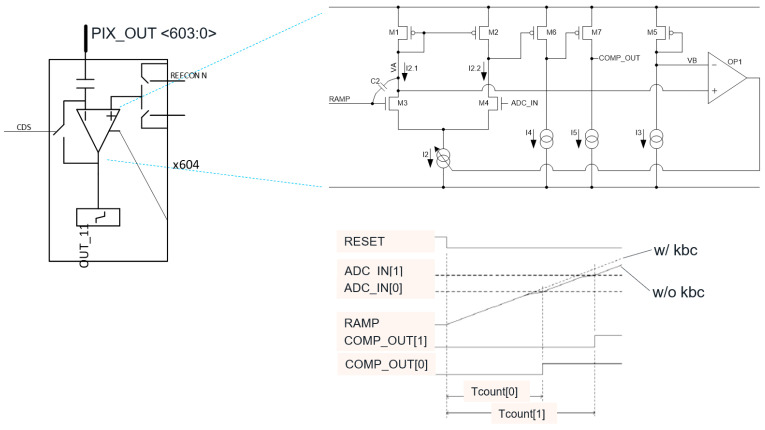
Analog part of the ramp ADC using the embedded kickback cancellation circuit, which reduces the area of the ramp capacitance by a factor of four times.

The simplified circuitry of the analog readout part is depicted in Figure 5. The column bias circuitry was placed in the pixel silicon layer to spare the logic silicon area and to improve the noise performance. The traditional column sample and hold circuitry [11] were removed to reduce the area and improve noise performance, with column analog and digital correlated double sampling operations performed directly in the ramp ADC.

To reduce the area occupied by the normally large capacitor at the output of the ramp generator, we used our kickback cancellation (KBC) technique [13]. The simplified column circuitry is depicted in Figure 5. The column of the pixel (PIX_OUT signal) is connected to the input stage of the ADC via column-level HB. The comparator of the ADC contains feedback loop circuitry as shown on the right of Figure 5. The bottom right of Figure 5 shows that each toggling comparator shifts the ramp signal. This shift is proportional to the ratio between C2 and the total ramp capacitance. The feedback loop changes the bias current of the input stage of the comparator to keep node VA constant, attenuating the kickback and compensating the ramp shift, as shown in the bottom right of Figure 5. This technique allowed us to reduce the equivalent column capacitance needed by the ramp generator by a factor of four times at the same kickback performance. This nearly halved the footprint of the analog readout portion of the sensor. The impact could be even greater, depending on the capacitor density available in the technology node.

### 2.2. Power Consumption Reduction

We made extensive use of digital-friendly readout circuits [14], starting with the ramp ADC, which contains minimal analog circuitry. The scaled technology node (40 nm) of the logic layer enabled dramatic power reductions in the readout compared to our previous designs in older technology nodes. Certain building blocks that were the most power-hungry in our previous designs (e.g., ADC counter) experienced an order-of-magnitude reduction in power consumption. Additionally, we used advanced power-down techniques to optimize current consumption by turning on specific blocks only when needed. As shown in Table 1, the new power-down modes enabled power consumption values that scale nearly linearly with the frame rate. This is achieved by turning off all readout blocks as soon as a frame has been sent off-chip. For instance, at 30 fps, the frame time is ~33 ms but the frame readout time is 5 ms (equivalent to the maximum frame rate of 200 fps). It follows that the readout portion of the sensor can be kept off for ~28 ms (33–5 ms).

## 3. HDR Operation

One of the main disadvantages of global shutter pixels, especially voltage domain ones, is the difficulty in achieving high dynamic range (HDR) at a reasonable pixel pitch. Some of the recent attempts include pixel-level ADC [15], dual gain [16,17], and LOFIC [18,19]. The pixel-level ADC method is equivalent to a global shutter and can relatively easily implement HDR by using the common dual quantization technique [20]. This, however, comes at the cost of a very large pixel pitch. Even with 3D stacking technology, the pixel pitch stays above 4 µm [15]. Dual gain, LOFIC, and all other HDR approaches that rely on a low-sensitivity signal and high-sensitivity signal combination require the doubling of the in-pixel storage capacitors, increasing the pixel pitch to at least 3.45 µm [16,17].

**Figure 6:**
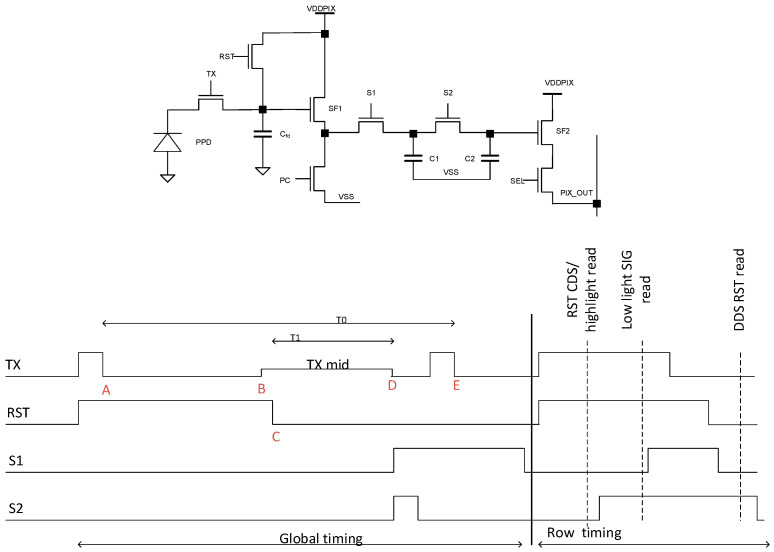
Voltage-domain GS pixel and simplified HDR timing. C2 stores overflow signal (at high light) or reset signal (at low light). The reset level of overflow is read during row readout. Only two capacitors are then needed to store the reset low light, low-light signal, overflow signal, and reset of overflow. CDS at low light is maintained, allowing low noise in dark conditions. Overflow happens during short T1 at FD, avoiding the need for a large overflow cap and reducing DSNU and dark current.

To achieve HDR with a small pixel pitch, we developed a new method to read high-light and low-light signals and their reset levels by using only two storage capacitors per pixel [6]. In this work, we use the traditional 8T pixel (Figure 6), which does not include dual gain operation. To validate the reduction in storage capacitor idea with the current pixel, we combined LOFIC and dual exposure into the charge-overflow-on-FD technique shown in Figure 6.

Overflow operation is done on the floating diffusion node (FD), at a fraction (T1) of the low-light exposure time (T0), with the prior period (T0–T1) having FD in reset. The DR is extended by the classic T0/T1 ratio, with T0 exploiting the entire full well of the photodiode and T1 exploiting the entire full well of floating diffusion.

The technique makes use of the timing diagram of Figure 6 and works as follows. (A) Long exposure T0 starts when TX toggles low. (B) TX toggles high- to mid-level. This facilitates charge overflow from PD to FD in case of medium or high light. Pulsing TX to mid-level right before T1 starts ensures that overflowing charges before the start of T1 are discarded since the FD is in the reset state. This allows a linear HDR reconstruction. (C) Short exposure T1 starts when the reset switch is turned off. At this point, any overflowing charge is collected on the FD node. Since T1 is much shorter than T0 (e.g., 15x shorter), the FD leakage affecting T1 and the dark current affecting T0 are negligible. (D) T1 exposure ends when TX toggles back to the lowest level. T1 exposure is stored via S1 and S2 in C2. (E) Finally, T0 exposure ends when TX toggles high. The T0 signal is then stored on C1 via the S1 switch.

**Figure 7:**
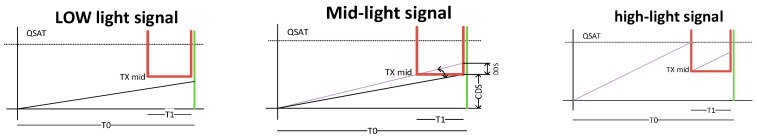
During low-light conditions (**left** plot), no signal overflows during T1. During mid-light conditions (**mid** plot), overflow happens just after T1 starts. During high-light conditions (**right** plot), overflow happens before T1 starts.

From above, the two storage capacitors store only the signal levels of T1 and T0 exposures. This would normally severely deteriorate the performance of the pixel due to the lack of CDS. However, we enable CDS for the low light signal (T0 exposure) and delta double sampling (DDS) for the high light signal (T1 exposure), as follows. Figure 7 shows three cases of light conditions (low, medium, and high light).

In the case of low-light conditions, no overflow occurs. Therefore, T1 exposure stored on C2 is the correlated reset level for long exposure T0, allowing CDS and hence keeping the noise level low (~5 e^−^) and the fixed pattern noise (FPN) under control (~3 e^−^).

In the case of a medium light level, overflow starts happening just after T1 starts (Figure 7, middle plot). To reconstruct the HDR image, both CDS (C2 − C1 signals) and DDS (C2 signal − dark reference) are needed during medium light since, as seen in the middle plot, their addition is the linear signal output. The choice of using both CDS and DDS is very important as it relaxes the process control requirements on the charge level of the overflow start. For instance, if the target overflow level is 5000 e^−^, the mid-light level can be defined starting at e.g., 3000 e^−^. When the light level is between 3000 e^−^ and 5000 e^−^, the DDS readout will not provide any signal in most sensors, with the CDS readout being sufficient. However, this choice would considerably increase the margin for a process variation of the overflow level, improving the yield of the sensor since the overflow level can deviate on the low side by up to 2000 e^−^ (5000 e^−^ target overflow − 3000 e^−^ mid-level definition) without impacting the HDR operation of the sensor. The drawback of this increased process margin is an increase in the SNR dip at mid-light levels.

The dark reference to allow for DDS is achieved during row readout by accessing the reset level of the pixel after reading the C2 and C1 signals, as shown in the timing diagram of Figure 6.

In the case of high light (Figure 7, right plot), the T0 exposure signal starts overflowing before T1 starts. Therefore, the CDS signal does not provide any meaningful information since it is not known when the overflow started. This means that in the case of high light, only the T1 signal is used for the HDR reconstruction. Therefore, DDS is used and CDS is discarded.

**Figure 8:**
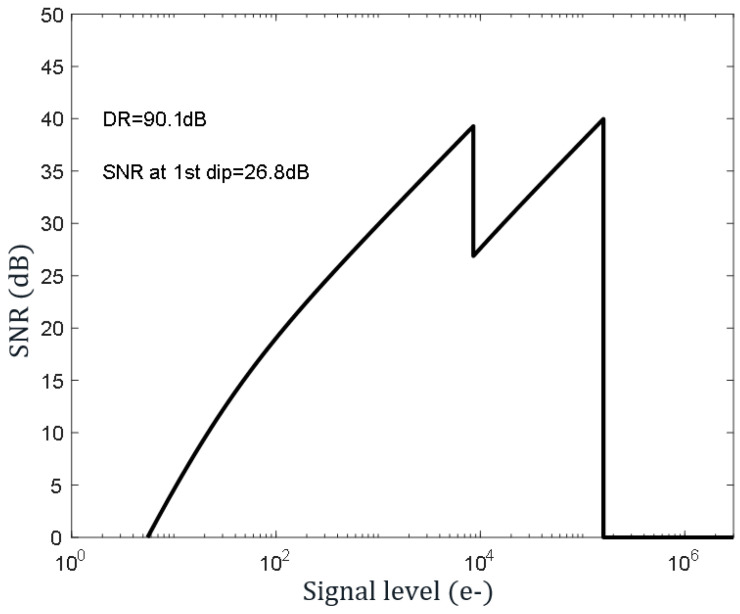
~90 dB DR is achieved with T0/T1 = 16x. A larger DR is possible by trading off with an SNR dip. Part of the data from the plot is simulated as a full char report and is not available for the current product in HDR mode.

With the technique above, only two in-pixel capacitors are necessary for the four signals needed for HDR (low light + reset, high light + reset), as compared to the four capacitors needed by [16], for example. The main novelty of this technique is that CDS is maintained at low light, despite using only two capacitors, as explained in the low-light operation of the paragraph above. This method, employing only two capacitors for four signals, can be used with many other HDR techniques, including conventional LOFIC, dual exposure, dual gain, etc. Contrary to alternative HDR techniques, which use skimming [21,22], the proposed method does not need FPN calibration, and pipeline readout is possible, improving low-light performance. Figure 8 shows ~90.1 dB DR with ~26.8 dB at the dip point, which is sufficient for many consumer applications such as eye tracking and hand/head tracking.

**Figure 9:**
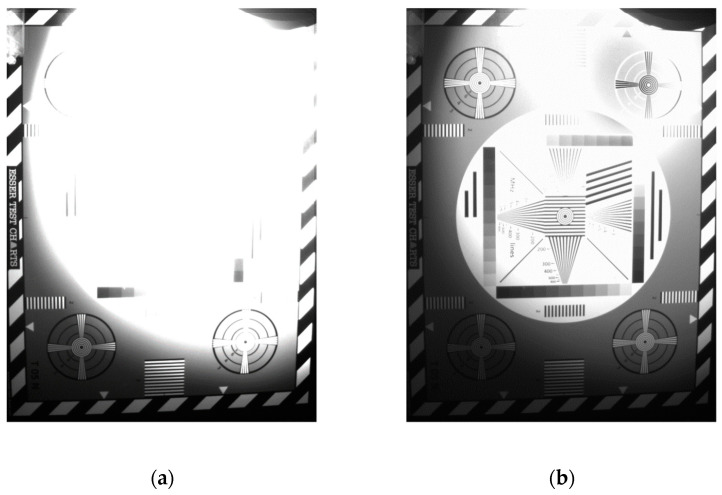
Image captures at 12-bit 1× gain with same light intensity at 1 ms exposure. (**a**) Standard mode; (**b**) HDR mode.

Figure 9 shows an image captured in standard 12b 1× gain mode compared to the same bit mode but with HDR operation active. The light source used in this experiment was a xenon lamp. As can be seen, the highlighted part is recovered thanks to the shorter T1 exposure. Since the HDR technique was developed post-design, very limited HDR functionalities are testable with the current silicon. Extended HDR measurements with the current technique will be available in a revised chip design.

## 4. Event Detection Mode

**Figure 10:**
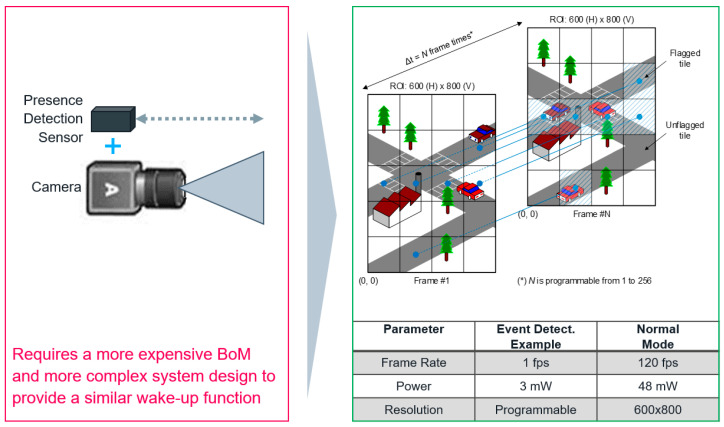
User-programmable event detection mode operating at 1 fps. When an event is detected, the sensor switches to another user-defined mode (e.g., 120 fps, 10 bits, 0.5 MP).

The sensor includes an event detection mode for low-power operation when no movement is happening in the scene. As shown in Figure 10, the sensor splits the pixel array into a programmable number of tiles composed of binned and/or subsampled pixels and runs at 1 fps with low power (<3 mW). The tile values are compared to their previous value every N frames (programmable). When a certain threshold is crossed, the event detection is triggered, which can initiate an automatic context switch in the sensor.

The time interval of comparing the tile values depends on the nature of the event of interest. Short intervals (e.g., every frame) allow the detection of short events like a fast car movement but increase the likeliness of false positives. Long intervals (e.g., every N frames with N > 2) allow the detection of longer events like a person walking by. The threshold determines the sensitivity of the event detection. If the on-chip algorithm detects an event, the sensor switches to a user-defined operation mode (e.g., 120 fps, 10 bits). Compared to the ~48 mW of power consumed in 120 fps mode, this method allows a drastic reduction in power consumption for some applications where the sensor is placed on non-moving cameras, such as computing, doorbells, security/surveillance, etc.

## 5. Conclusions

**Figure 11:**
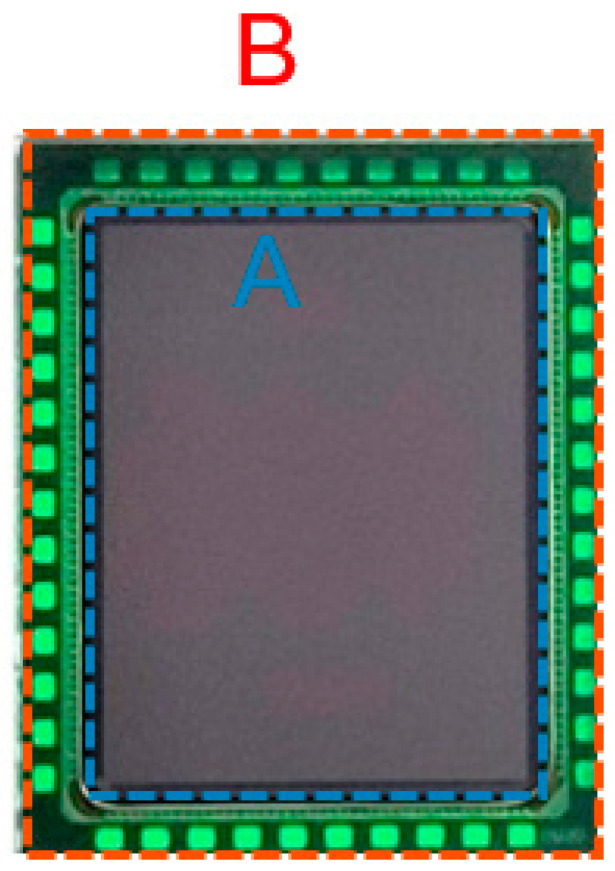
A 2.3 mm × 2.8 mm CSP version where A and B denote the optical size and the total package size, respectively.

With a 2.3 mm × 2.8 mm packaged size (Figure 11), the presented sensor is one of the smallest GS sensors reported in the literature and is commercially available. Despite its size, it contains 0.5 MP, a MIPI CSI2 interface, ultra-low power operation, and smart event detection modes, and proves the single-shot HDR concept with pipeline operation. The specialty of the proposed HDR technique is that it reuses the same pixel storage capacitor for both reset levels of low-light conditions and signal levels of high-light conditions.

Combined with QE enhancement technology, made possible by BSI technology (>92% in the visible wavelength, >36% in the NIR 940 nm range) it is comparable to past works [7,10,16,17] in terms of electro-optical performance, as shown in Table 2, while it excels in the top specifications of AR/VR applications, being low power and small footprint. It consumes <20 mW at 30 fps and <48 mW at 120 fps. In terms of area efficiency, it outperforms the listed state-of-the-art works with its 58% ratio between optical and overall package area, being an ideal candidate for consumer AR/VR devices. With better area efficiency, we could fit more and larger pixels compared to, e.g., [10], improving on both resolution and sensitivity.

## Figures and Tables

**Table 1:** Power consumption of the sensor at different fps.

Mode	Power Consumption
30 fps, 10 bits	18.5 mW
60 fps, 10 bits	30 mW
120 fps, 10 bits	48 mW
200 fps, 10 bits	75 mW

**Table 2:** Comparison table.

Parameter	[7]	[10]	[17]	[16]	This Work
technology	Triple-Stacked 65 nm + 65 nm + 45 nm	stacked 45 nm − 65 nm	stacked 45 nm − 65 nm	stacked 45 nm − 65 nm	**stacked** **45 nm + 40 nm**
Pixel pitch (µm)	1.8	2.2	4	3.45	**2.79**
Resolution	1280 × 1024	640 × 480	1024 × 832	5 MP	**600 × 800**
Shutter	Global VD	Global VD	Global VD	Global VD	**Global VD**
DR (dB)	68 (HCG)	61	90	90	**90**
Noise (e−)	1.8 (HCG)	2.3 (HCG mode)	4	2.7 (HCG)	**5 (LCG mode)**
Power (mW)	-	139	-	-	**<20 mW @10 b, ** **30 fps, ** **48 mW @120 fps**
Footprint efficiency ratio (%) *	-	19.2	21.3	-	**58**
Footprint (mm × mm)	-	2.6 × 2.95	8 × 8	-	**2.3 × 2.8**
QE 940 nm (%)	Only visible reported	38	40	41	**36**

* Represents ratio between optical area and overall die area (A/B of Figure 11).

## Data Availability

Data are contained within the article.
